# Supplementary material for: Chemical composition of isoprene SOA under acidic and non-acidic conditions: effect of relative humidity
Source: Atmos Chem Phys. Author manuscript; Available in PMC 2020 Mar 10. (PMC7063744; doi:10.5194/acp-18-18101-2018)
Supplement: Supp Info [file NIHMS1045602-supplement-Supp_Info.pdf]

Supplement of Atmos. Chem. Phys., 18, 18101–18121, 2018
https://doi.org/10.5194/acp-18-18101-2018-supplement
© Author(s) 2018. This work is distributed under
the Creative Commons Attribution 4.0 License.

Supplement of

Chemical composition of isoprene SOA under acidic and non-acidic conditions: effect of relative humidity

Klara Nestorowicz et al.

Correspondence to: Rafal Szmigielski (ralf@ichf.edu.pl) and Mohammed Jaoui (jaoui.mohammed@epa.gov)

The copyright of individual parts of the supplement might differ from the CC BY 4.0 License.

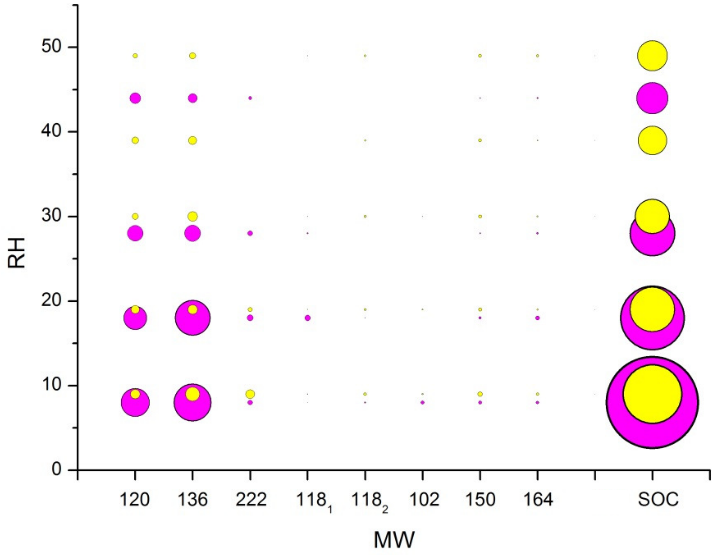

Figure S1. Relative amounts of aerosol components detected with GC-MS acidic seed (pink) and non-acidic seed (yellow) experiments (the areas of the circles are proportional to the estimated mass concentrations of compounds).

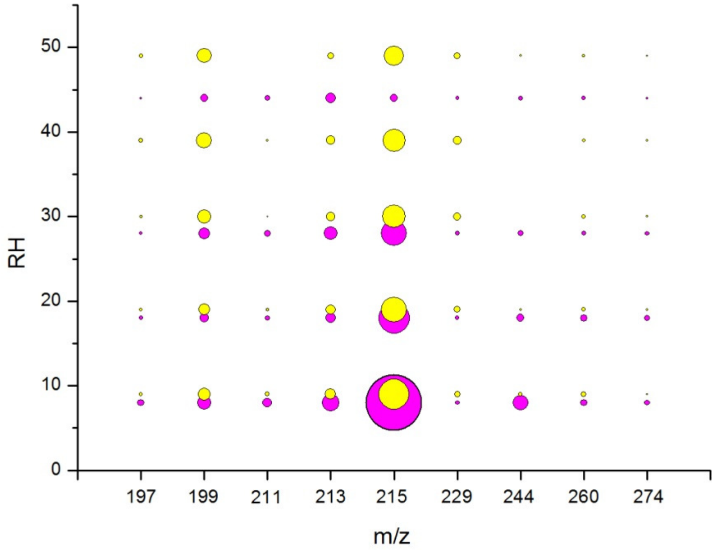

Figure S2. Relative abundances of aerosol components detected with LC-MS in acidic seed (pink) and non-acidic seed (yellow) experiments (the areas of the circles are proportional to relative abundances of compounds detected).

Table S1. Comparison of product yields in acidic seed experiments vs. non-acidic seed experiments at various RH levels (> higher, = equal and lower <)

<table>
<tr><th>Product</th><th>MW</th><th>m/z</th><th>RH = 8 – 9</th><th>RH = 18 – 20</th><th>RH = 28 – 30</th><th>RH = 39 – 49</th></tr>
<tr><td>2-methylglyceric acid</td><td>120</td><td></td><td colspan="4">></td></tr>
<tr><td>2-methyltetrol OS</td><td></td><td>244</td><td colspan="4">></td></tr>
<tr><td>2-methylthreonic acid NOS</td><td></td><td>274</td><td colspan="4">></td></tr>
<tr><td>furanone OS</td><td></td><td>211</td><td colspan="4">></td></tr>
<tr><td>2-methyltetrols</td><td>136</td><td></td><td colspan="3">></td><td>=</td></tr>
<tr><td>2-methyltetrol NOS</td><td></td><td>260</td><td colspan="2">></td><td colspan="2">=</td></tr>
<tr><td>furanetriol OS</td><td></td><td>213</td><td>></td><td colspan="3">=</td></tr>
<tr><td>2-methyltetrol OS</td><td></td><td>215</td><td>></td><td colspan="3">=</td></tr>
<tr><td>IEPOX-1</td><td>118</td><td></td><td>=</td><td>></td><td colspan="2">=</td></tr>
<tr><td>dimer of 2-methylglyceric acid</td><td>222</td><td></td><td><</td><td colspan="3">></td></tr>
<tr><td>C5-diol</td><td>102</td><td></td><td>></td><td><</td><td colspan="2">=</td></tr>
<tr><td>IEPOX OS</td><td></td><td>197</td><td>></td><td colspan="2">=</td><td><</td></tr>
<tr><td>2-methylglyceric acid OS</td><td></td><td>199</td><td>=</td><td colspan="3"><</td></tr>
<tr><td>IEPOX-2</td><td>118</td><td></td><td colspan="4"><</td></tr>
<tr><td>2-methylthreonic acid OS</td><td></td><td>229</td><td colspan="4"><</td></tr>
</table>

Figure S3. Concentrations or relative abundances of some compounds in acidic seed experiments (blue) and non-acidic seed experiments (red) – influence of Relative Humidity

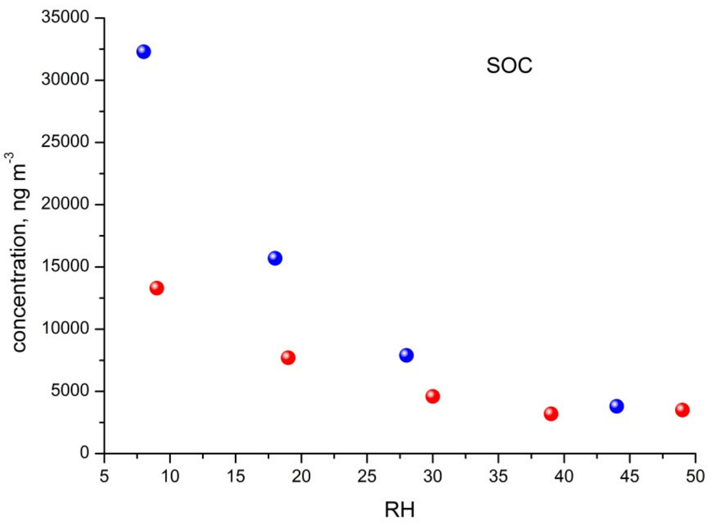

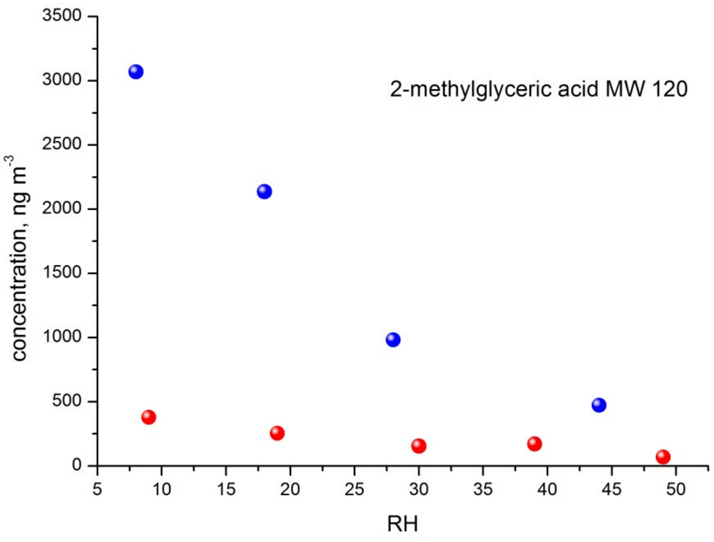

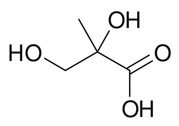

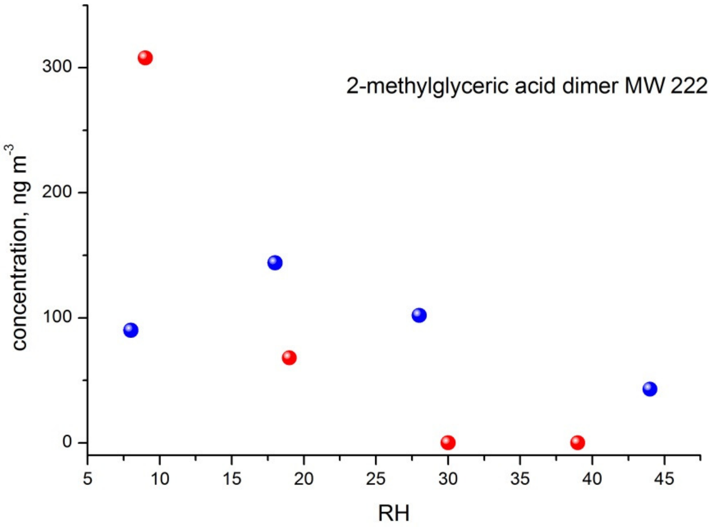

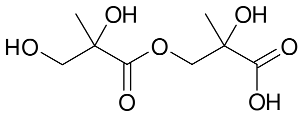

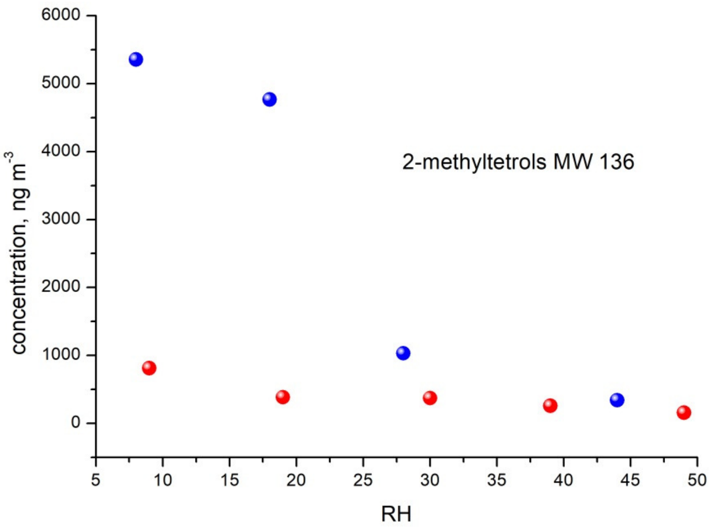

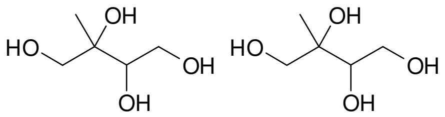

2-methylthreitol (left) and 2-methylerythritol (right)

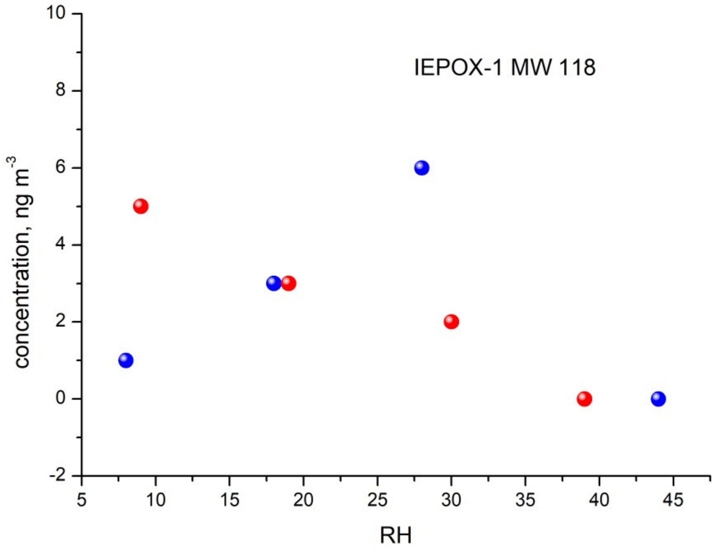
IEPOX-1 MW 118
concentration, ng m-3
RH
10
8
6
4
2
0
-2
5
10
15
20
25
30
35
40
45

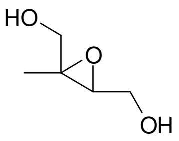
HO
O
OH

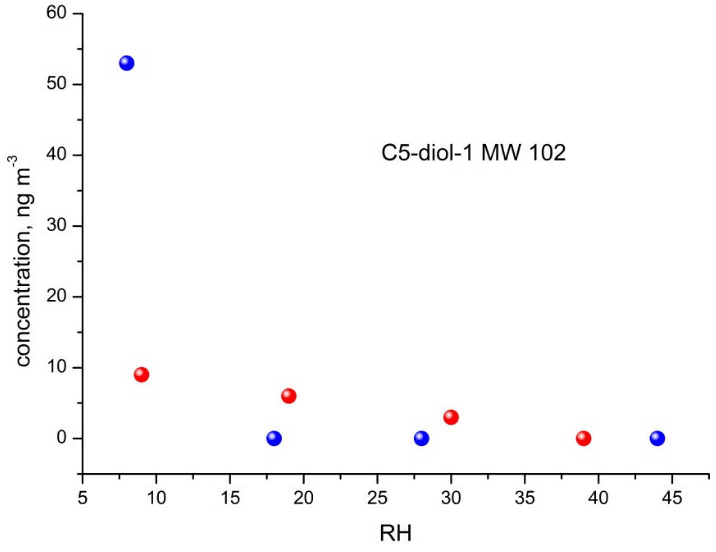
C5-diol-1 MW 102
concentration, ng m-3
RH
60
50
40
30
20
10
0
5
10
15
20
25
30
35
40
45

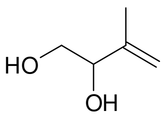
HO
OH

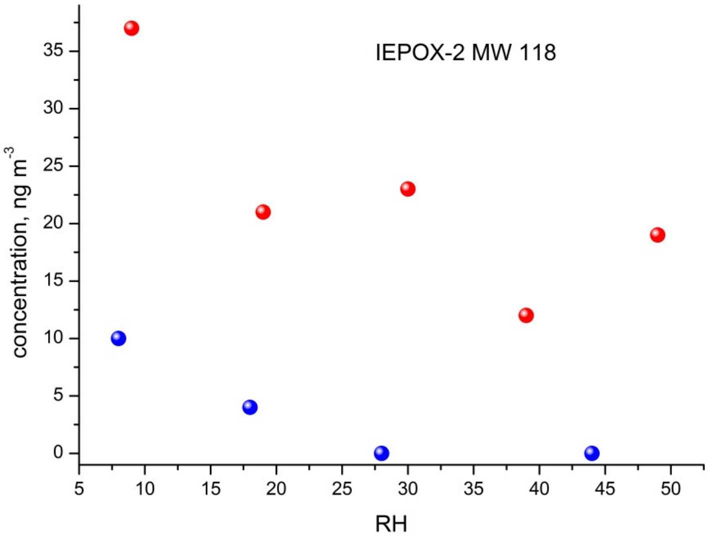
IEPOX-2 MW 118
concentration, ng m-3
RH
0
5
10
15
20
25
30
35
40
45
50

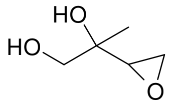
HO
HO
O

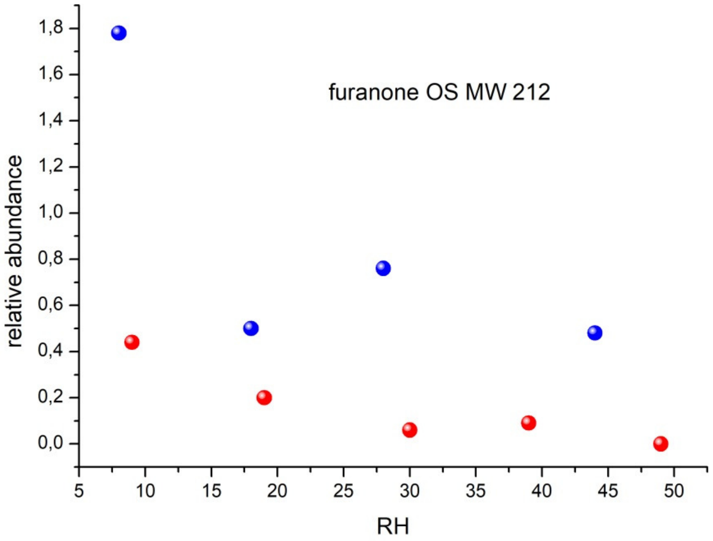
furanone OS MW 212
relative abundance
RH
0,0
0,2
0,4
0,6
0,8
1,0
1,2
1,4
1,6
1,8
5
10
15
20
25
30
35
40
45
50

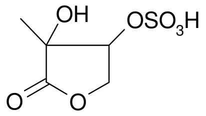
OH
OSO3H
O
O

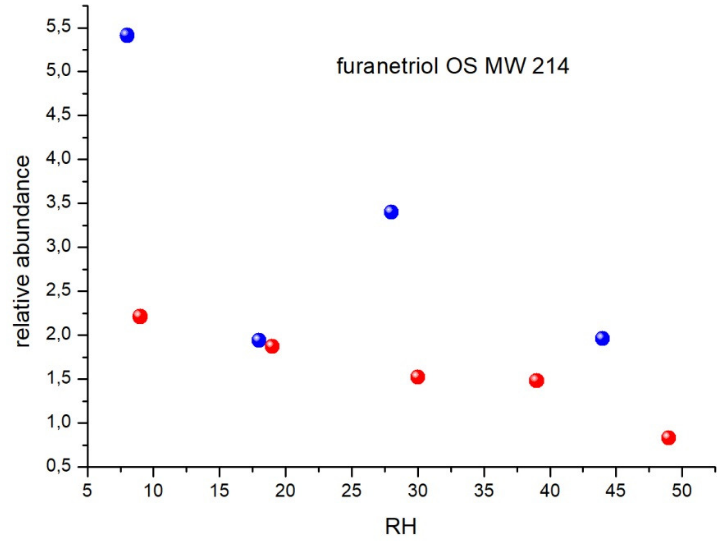
furanetriol OS MW 214
relative abundance
RH
5,5
5,0
4,5
4,0
3,5
3,0
2,5
2,0
1,5
1,0
0,5
5
10
15
20
25
30
35
40
45
50

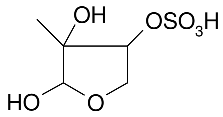
OH
OSO3H
HO
O

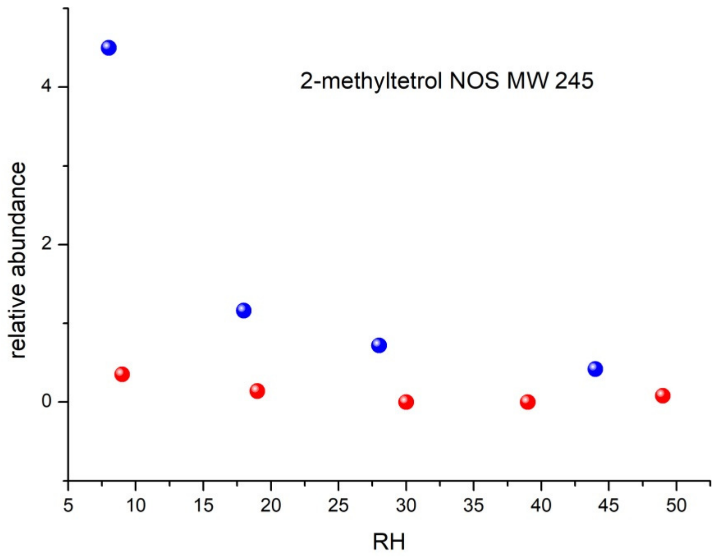
2-methyltetrol NOS MW 245
relative abundance
RH
4
2
0
5
10
15
20
25
30
35
40
45
50

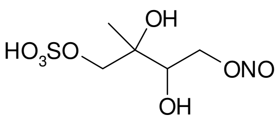
OH
HO3SO
ONO
OH

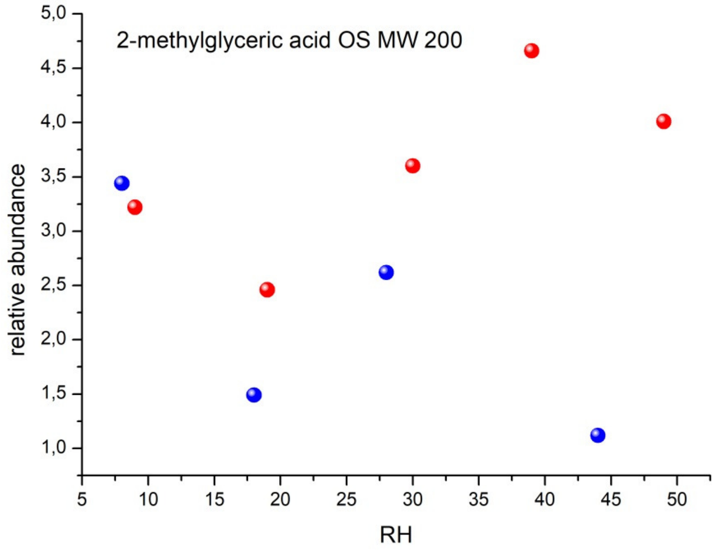
2-methylglyceric acid OS MW 200
relative abundance
RH
5,0
4,5
4,0
3,5
3,0
2,5
2,0
1,5
1,0
5
10
15
20
25
30
35
40
45
50

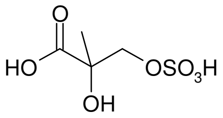
O
HO
OSO3H
OH

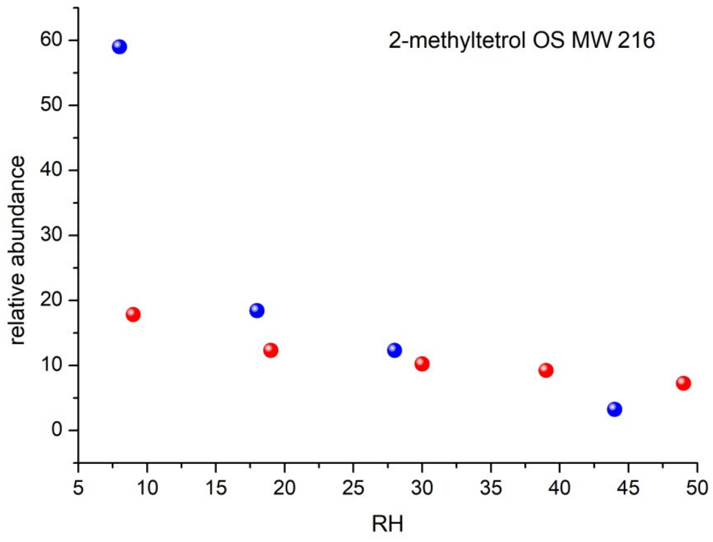
2-methyltetrol OS MW 216
relative abundance
RH
60
50
40
30
20
10
0
5
10
15
20
25
30
35
40
45
50

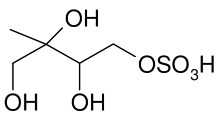
OH
OSO3H
OH
OH

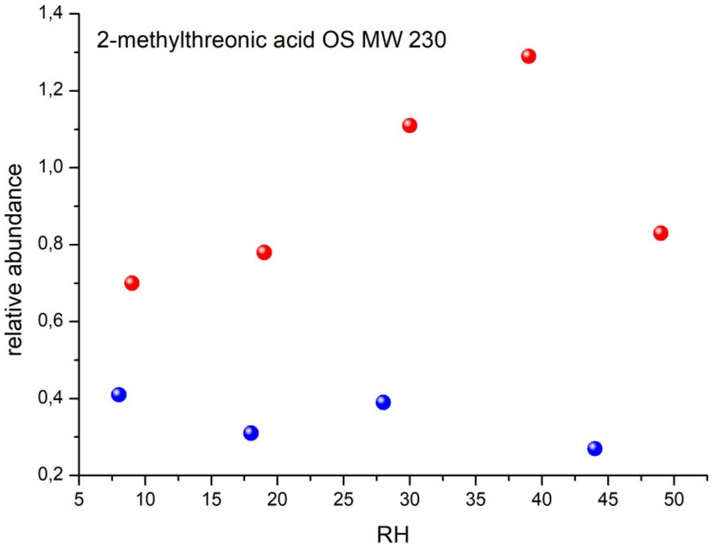
2-methylthreonic acid OS MW 230
relative abundance
RH
1,4
1,2
1,0
0,8
0,6
0,4
0,2
5 10 15 20 25 30 35 40 45 50

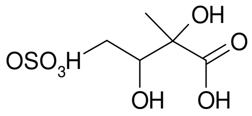
OSO3H
OH
O
OH
OH

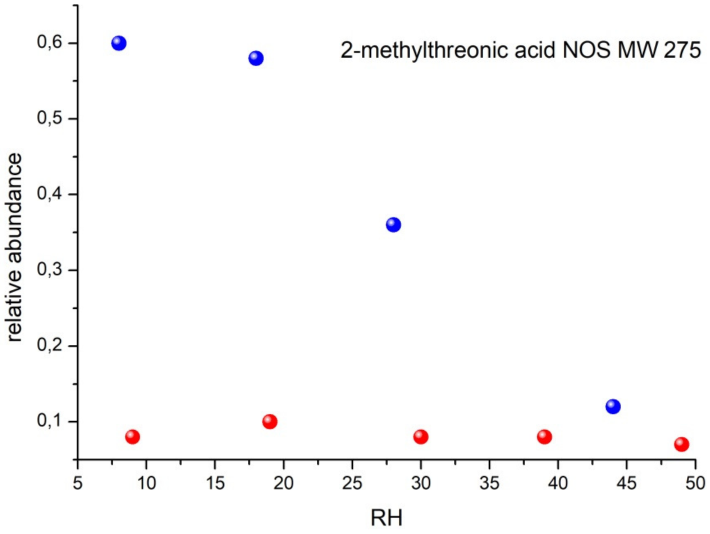
2-methylthreonic acid NOS MW 275
relative abundance
RH
0,6
0,5
0,4
0,3
0,2
0,1
5 10 15 20 25 30 35 40 45 50

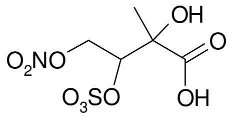
O2NO
OH
O
O3SO
OH

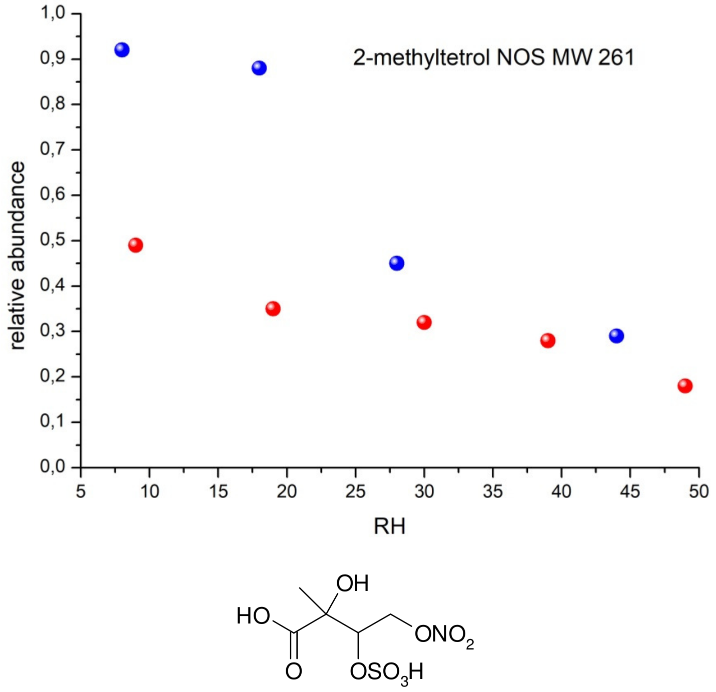
2-methyltetrol NOS MW 261
relative abundance
RH
0,0
0,1
0,2
0,3
0,4
0,5
0,6
0,7
0,8
0,9
1,0
5
10
15
20
25
30
35
40
45
50
OH
HO
ONO2
O
OSO3H

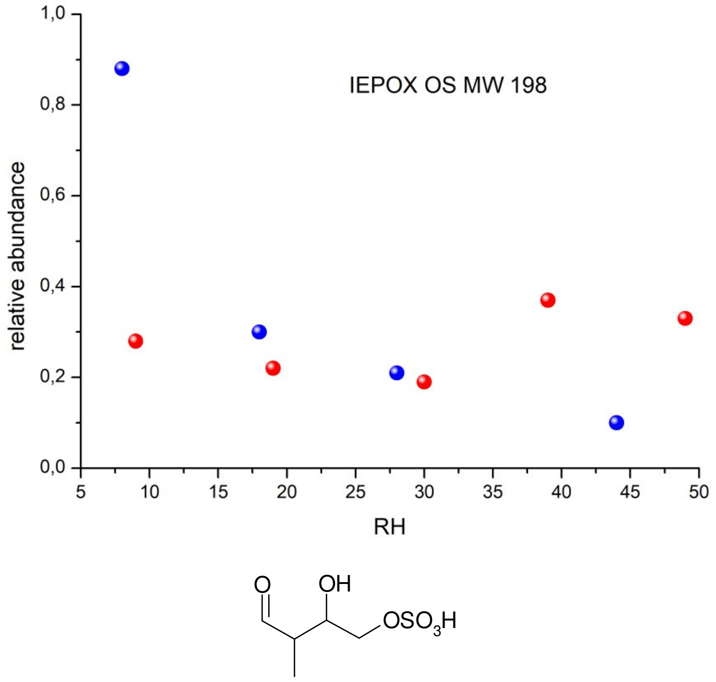
IEPOX OS MW 198
relative abundance
RH
0,0
0,2
0,4
0,6
0,8
1,0
5
10
15
20
25
30
35
40
45
50
O
OH
OSO3H

Figure S4. Extracted Ion Chromatograms (EIC) of selected components detected in the respective filter extracts from smog chamber ISO SOA (ER667 – non-acidic seed aerosol; ER662 – acidic seed aerosol) and $PM_{2.5}$ ambient summer aerosol from Godow and Zielonka sites.

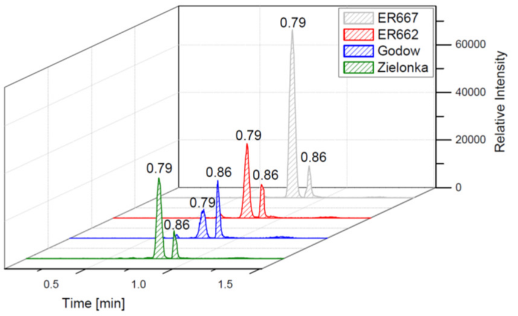

2-methyltetrol organosulfate (MW 216)

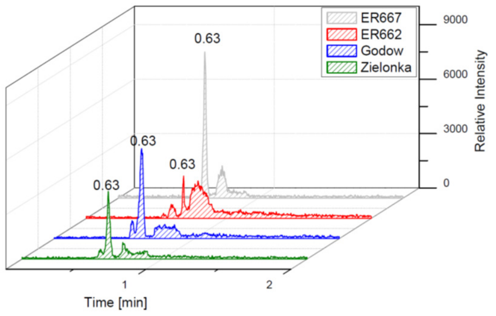

2-methylthreonic acid organosulfate (MW 230)

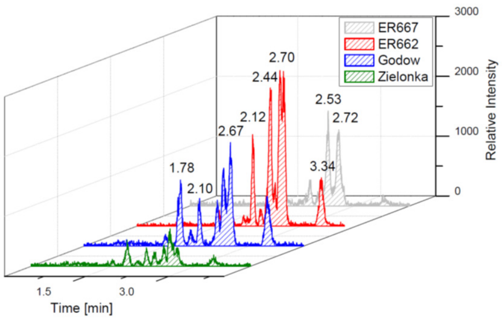

2-methyltetrol nitroxy-organosulfate
(MW 261)

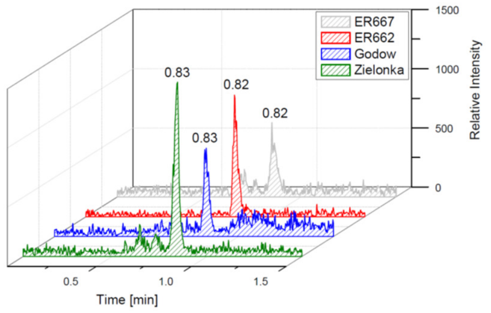

2-methylthreonic acid nitroxy-organosulfate
(MW 275)
